# ﻿Taxonomic clarifications, a new combination, and three new species of Neotropical *Rinorea* (Violaceae)

**DOI:** 10.3897/phytokeys.256.144950

**Published:** 2025-05-29

**Authors:** Saúl E. Hoyos-Gómez, Harvey E. Ballard Jr., Ricardo Callejas Posada, Gregory A. Wahlert

**Affiliations:** 1 Universidad de Antioquia, Instituto de Biología, Apartado postal 1226, Medellín, Colombia Universidad de Antioquia Medellín Colombia; 2 Department of Environmental and Plant Biology, Ohio University, Athens, Ohio, 45701, USA Ohio University Athens United States of America; 3 Cheadle Center for Biodiversity and Ecological Restoration, University of California, Santa Barbara, Santa Barbara, California, 93106, USA University of California, Santa Barbara Santa Barbara United States of America

**Keywords:** Conservation, Neotropics, new species, *Rinorea* sect. *Pubiflorae*, *Rinorea* sect. *Rinorea*, taxonomy, Violaceae

## Abstract

Based on findings of revisionary studies of Neotropical *Rinorea*, we propose several taxonomic novelties and clarifications in this study, including the description of three new species, a new combination, the placement of three species in synonymy, and updated descriptions of two poorly known species from South America. One of the new species, *Rinoreaidarragae*, is described from Mesoamerica and belongs to R.sect.Rinorea. The two other new species are from South America and are placed in R.sect.Pubiflorae: *Rinoreacardenasii* occurs in the region of the western edge of the Guiana Shield in Colombia, and *Rinoreapabongonzaleziorum* is from the Amazon Basin in Colombia and Venezuela. Rinorealindenianavar.fernandeziana is elevated to the rank of species, and *Rinoreacordata*, *R.antioquiensis*, and *R.hymenosepala* are synonymized under *R.laurifolia*, *R.squamata*, and *R.ulmifolia*, respectively. Finally, we provide updated descriptions of *Rinoreadeflexa* from Ecuador and Peru and *Rinoreabicornuta* from Colombia and Brazil. For the three new species, we provide descriptions, illustrations, distribution maps, and preliminary conservation assessments based on IUCN Red List categories and criteria.

## ﻿Introduction

*Rinorea* Aubl. is the second largest genus in the Violaceae after *Viola*. In the Neotropics, the genus is composed of 54 species and three infraspecific taxa (Hekking 1988; Wahlert and Ballard 2009; Silva and Medeiros 2012; Wahlert and Ballard 2012; Ballard et al. 2014; Oliveira and Queiroz 2020). Taxonomic revision of *Rinorea* in Colombia and Mesoamerica has led to the discovery of several new species and segregates from across the Neotropics, resulting in the need for some new combinations and synonymies (Hoyos-Gómez et al. 2024).

## ﻿Materials and methods

This study is based on the examination of new collections made in Colombia and Costa Rica by SEHG and examination of herbarium material from the following herbaria: BHO, COAH, COL, ECUAMZ, F, FAUC, GH, HUA, JAUM, MO, NY, UBDC, and US (herbarium abbreviations follow Thiers 2024). Several digitized herbarium collections and records from the Global Biodiversity Information Facility (GBIF) were consulted to locate duplicate specimens for the following herbaria: CM, FMB, INPA, K, MBM, RB, P, U, and VEN. All cited collections have been seen by the first author. Species descriptions are based on field observations and herbarium specimens. Flowers from herbarium specimens were rehydrated and dissected to obtain accurate measurements using a dissecting stereoscope.

Post-facto geocoordinates for specimens lacking coordinates were assigned using either Google Earth or Tropicos specimen records with the same collecting localities. Collections lacking unambiguous locality information were omitted from the conservation assessment calculations. The online GeoCat facility (Bachman et al. 2011) was used to calculate the Extent of Occurrence (**EOO**) and Area of Occupancy (**AOO**) of each species to preliminarily estimate the risk of extinction using IUCN red list categories and criteria (IUCN 2014); a 2 × 2 km cell was used for calculating AOO.

## ﻿Taxonomic treatment

### ﻿New species

#### ﻿1. Rinorea cardenasii

Taxon classificationPlantaeMalpighialesViolaceae

Hoyos-Gómez sp. nov.

7CC9BAF4-9BF4-5C9E-8AFB-1A77CAC8F216

urn:lsid:ipni.org:names:77362478-1

##### Type.

Colombia • Dept. Guaviare: Mpio. San José del Guaviare, Serranía de la Lindosa, vereda Cerro Azul, camino desde la casa de José Noe por la trocha hacia el Raudal, 02°31'35"N, 72°52'16"W, 200–220 m, 20 Sep 2023, *S. E. Hoyos-Gómez et al. 5393* (holotype: HUA [acc. no. 236209; barcode HUA0049795]!; isotype: HUA [acc. no. 236210; barcode HUA0049796]!).

##### Diagnosis.

*Rinoreacardenasii* is similar to *Rinoreamacrocarpa* (Mart. ex Eichler) Kuntze by the opposite leaves and racemose inflorescence, but it differs by the obovate lamina (vs. elliptic lamina in *R.macrocarpa*), 4 to 6 major secondary vein pairs (vs. 7 to 9), domatia present (vs. absent), connective scales with entire margin (vs. praemorse margin), filaments free (vs. filaments connate at the base forming a staminal tube), fruit 2.5–3.2 × 2–2.5 cm (vs. 3.5–5.8 × 2.5–3 cm), two pubescent seeds per valve (vs. three glabrous seeds), and seeds 5–7 mm in diam. (vs. 7.6–9 mm in diam.).

##### Description.

**Figure 1.**
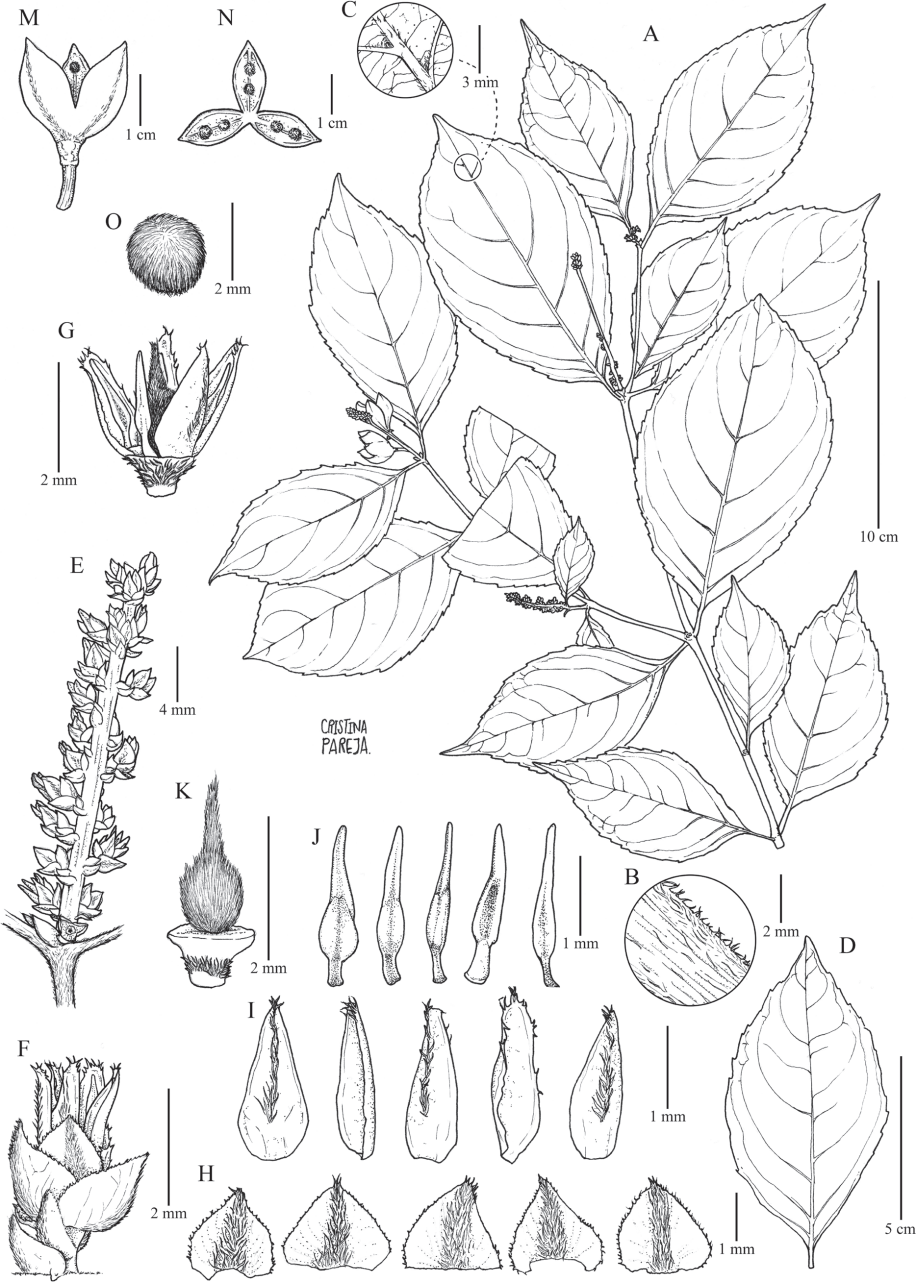
*Rinoreacardenasii* Hoyos-Gómez **A** habit **B** detail of pubescence on young twig **C** detail leaf domatia, abaxial surface **D** leaf architecture **E** inflorescence **F** flower **G** detail of flower showing free filaments, petals, and gynoecium **H** sepals, abaxial surface **I** petals showing detail of abaxial and adaxial surfaces **J** stamens showing detail of abaxial and adaxial surfaces **K** gynoecium **M** fruit **N** detail of fruit showing two seeds per carpel **O** seed (**A–O**: *H. Mendoza et al. 11676* [JAUM]).

Treelets 3–4 m tall, terminal branchlets pubescent with dimorphic ferruginous pubescence, small erect trichomes 0.2–0.3 mm long and longer appressed trichomes 0.4–0.6 mm long. Leaves opposite, petiolate; petiole 3–7 cm, pubescent with dimorphic ferruginous pubescence, small erect trichomes 0.1–0.3 mm long and longer appressed trichomes 0.4–0.6 mm long; stipules deciduous, free, triangular, 3–6 × 1.3–1.8 mm, densely pubescent with appressed ferruginous trichomes 0.2–0.3 mm long; lamina obovate, 6–15 × 4–8 cm, foliaceous, veins abaxially pubescent with appressed ferruginous trichomes 0.3–0.5 mm long, veins pinnate, semicraspedodromous, with 4 to 6 pairs of major secondary vein, secondary veins with unequal spacing between them, percurrent, base symmetrical, cuneate, margin crenate and ciliolate with ferruginous trichomes 0.2–0.3 mm long, apex mucronate to acuminate, acumen 0.8–1.5(2) cm long, leaf domatia present. Inflorescence terminal, racemose, solitary, 3–5 × 0.5–1 cm, central axis brown, densely pubescent with erect ferruginous trichomes 0.2–0.3 mm long; pedicels 0.7–1.5 mm, pubescent with erect ferruginous trichomes 0.2–0.3 mm long, articulated at the base; bracts persistent, triangular, 1.5–2 × 1.2–1.5 mm, costa pubescent with appressed golden ferruginous trichomes 0.2–0.3 mm long, margin ciliolate, apex apiculate; bracteoles persistent, triangular, 1–1.2 × 0.7–0.9 mm, opposite, costate, pubescent with appressed trichomes 0.2–0.3 mm long, margin ciliolate with ferruginous trichomes, apex mucronulate. Flowers 3–4 × 3–4 mm, subzygomorphic; sepals triangular, 1.8–2 × 1.5–2 mm, with 5 to 9 longitudinal veins, pubescent with appressed golden trichomes 0.2–0.3 mm long, margin ciliolate, apex apiculate; petals white, lanceolate, 2.5–3 × 1–1.3 mm, costa pubescent with appressed ferruginous trichomes 0.3–0.5 mm long, margin ciliolate, apex acute; stamens 2.2–2.5 × 0.4–0.6 mm, filaments free, dorsal gland covering the basal part of the filament, sparsely pubescent with golden appressed trichomes 0.1–0.2 mm long, anthers ellipsoid, 0.5–0.7 × 0.4–0.5 mm, glabrous, connective 0.9–1 mm long, apically pubescent with 1 to 2 golden setae 0.3–0.5 mm long, glabrescent; dorsal anther connective scale lanceolate, 2–2.2 × 0.5–0.7 mm, margin entire, brown; ovary globose, 0.8–1 × 0.9–1 mm, pubescent with appressed golden trichomes 0.3–0.4 mm long; style filiform, erect, 1–1.2 mm long, pubescent with trichomes 0.2–0.3 mm long, stigma acute. Fruit a symmetrical subligneous capsule dehiscent along three sutures, ellipsoid, 1.5–2.5 × 1.5–2 cm, apex truncate, green when fresh, brown when dry, dimorphic golden pubescence with small erect trichomes 0.2–0.3 mm long and longer appressed trichomes 0.4–0.6 mm long. Seeds two per valve, globose, 3–4 mm diam., brown at maturity, densely pubescent with golden trichomes 0.3–0.5 mm long (Fig. 1).

##### Distribution and habitat.

**Figure 2.**
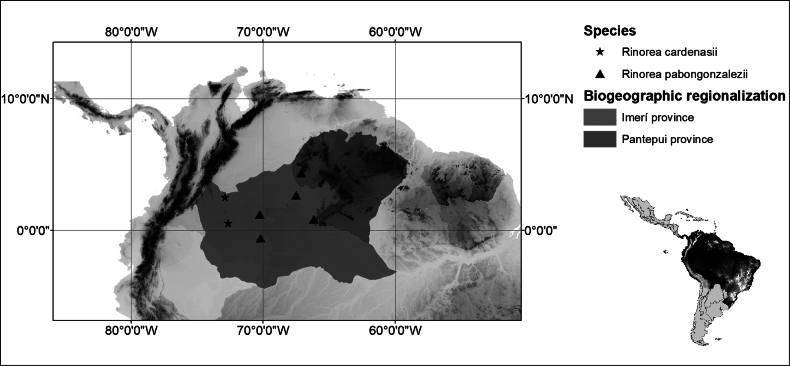
Distribution of *Rinoreacardenasii* (stars) and *R.pabongonzalezii* (triangles).

*Rinoreacardenasii* is an endemic species from the Departments of Caquetá and Guaviare in eastern Colombia, where it is restricted to areas of lowland forest at elevations of 200 to 350 m (Fig. 2). It grows in the vicinity of rocky outcrops of the Serranía de La Lindosa and the Serranía del Chiribiquete, which are the westernmost outcrops of the Guiana Shield in Colombia.

##### Etymology.

*Rinoreacardenasii* is named in honor of the late Dairon Cárdenas López (1957–2022) of the Instituto Amazónico de Investigaciones Científicas (SINCHI) and director of the COAH herbarium in Bogotá, Colombia. Cárdenas López made many significant contributions to the knowledge of the flora of Amazonian Colombia.

##### Phenology.

The species has been recorded with flowers and fruit in September.

##### Conservation status.

*Rinoreacardenasii* has an extremely limited distribution and is known from only three collections representing three occurrences and two subpopulations. It has a geographic range in the form of an estimated EOO of 8 km^2^ (adjusted upward from 3.017 km^2^ following IUCN guidelines, 2024) and an AOO of 8 km^2^. The three occurrences are located outside protected areas and are threatened by deforestation for timber extraction, agriculture, and grazing. With respect to the most serious plausible threat of deforestation, the three occurrences represent two locations, which falls within the limits for “Endangered” status. We infer an ongoing loss of habitat that will lead to continuing decline in AOO, EOO, habitat extent and quality, and the number of subpopulations and mature individuals. *Rinoreacardenasii* is therefore preliminarily assessed as “Endangered” [EN B1ab(i,ii,iii,iv,v)+2ab(i,ii,iii,iv,v)] in accordance with the IUCN Red List Categories and Criteria (IUCN 2024).

##### Notes.

*Rinoreacardenasii* is most similar morphologically to *R.macrocarpa* and *R.pabongonzaleziorum*. *Rinoreacardenasii* can be differentiated from *R.pabongonzaleziorum* by its symmetric, cuneate lamina base (vs. symmetric, decurrent lamina base in *R.pabongonzaleziorum*), its petals with a pubescent costa and ciliolate margin (vs. costa with scattered pubescence and entire margin), its pubescent sepals with 5–9 longitudinal veins (vs. densely pubescent sepals without longitudinal veins), its free filaments (vs. connate filaments forming a staminal tube), and its two pubescent seeds per valve (vs. one glabrous seed per valve). Table 1 highlights the key morphological differences among *Rinoreacardenasii*, *R.macrocarpa*, and *R.pabongonzaleziorum*.

**Table 1.** Key morphological differences among *Rinoreacardenasii*, *R.macrocarpa*, and *R.pabongonzaleziorum*.

Character	*R.cardenasii*	*R.macrocarpa*	*R.pabongonzaleziorum*
Lamina shape	ovate	Elliptic	obovate
Lamina base	symmetric, cuneate	symmetric, cuneate	symmetric, decurrent
Lamina pubescence	adaxial surface glabrous; abaxial surface with pubescent on veins	both surfaces minutely pubescence	adaxial surface glabrous; abaxial surface with pubescence on veins
Inflorescence length	3–5 cm	3–9 cm	3–4 cm
Petal pubescence	costa pubescent, margin ciliolate	costa glabrous, margin entire	costa with scattered pubescence, margin entire
Sepal pubescence	pubescent, 5 to 9 longitudinal veins	pubescent apically, no longitudinal veins	densely pubescent, no longitudinal veins
Filament connation	not connate	connate to form a staminal tube	connate to form a staminal tube
Seeds per valve	2	3	2
Seed pubescence	pubescent	glabrous	glabrous
Geographical distribution	Colombia	across Amazon Basin	Colombia, Venezuela

##### Additional specimens examined.

**Colombia. Dept. Caquetá**: • Mpio. Solano, Cuenca media del río Cuñare, bosque de tierra firme, 0°32'4"N, 72°37'58"W, 350 m, 15 Nov 2000 (fl), *H. Mendoza et al. 11676* (JAUM, FMB). **Dept. Guaviare**: • Mpio. San José del Guaviare, Serranía de la Lindosa, vereda Cerro Azul, camino por la trocha hacia el Raudal, 02°31'34"N, 72°52'15"W, 200 m, 13 Aug 2024 (fr), *S. E. Hoyos-Gómez et al. 5779* (HUA, JAUM, COAH, FMB).

#### ﻿2. Rinorea idarragae

Taxon classificationPlantaeMalpighialesViolaceae

Hoyos-Gómez sp. nov.

04A9E9EA-EDDB-5CAD-AE2C-1F57A54B6D85

urn:lsid:ipni.org:names:77362480-1

##### Type.

Costa Rica • Prov. Puntarenas: Reserva Forestal Golfo Dulce, Península de Osa, Rincón, Banegas, camino a Rancho Quemado en la primera cuesta, 08°40'34"N, 83°32'11"W, 192 m, 20 Jul 2010, *S. E. Hoyos-Gómez & R. A. Aguilar Fernández 1110* (holotype: MO [acc. no. 6335352; barcode MO-2326294]!; isotypes: MEDEL [acc. no. 56951]!; MO [acc. no. 6335353; barcode MO-2326291]!; MO [acc. no. 6603524; barcode MO-2679512]!; HUA [acc. no. 179370; barcode HUA–001977]!; HUA [acc. no. 179369; barcode HUA –0019778]!; JAUM [acc. no. 058851]!; JAUM [acc. no. 058843]!; CR [cat. no. 4238055]!; NY [barcode 02841133]!).

##### Diagnosis.

*Rinoreaidarragae* resembles *R.paniculata* (Mart.) Kuntze by the alternate leaves, elliptic lamina with symmetrical base, absence of domatia, thyrsoid inflorescence, filaments connate at the base forming a staminal tube, asymmetrical capsule pubescent with velutinous trichomes, and one glabrous seed per valve, but differs by the pubescent stipules (vs. glabrous), leaves brown and papery when dry (vs. leaves pale green with a dark brown margin and coriaceous), cymules with 7 to 10(15) flowers (vs. 3 to 4[7]), and style sigmoid at the base (vs. style straight).

##### Description.

**Figure 3.**
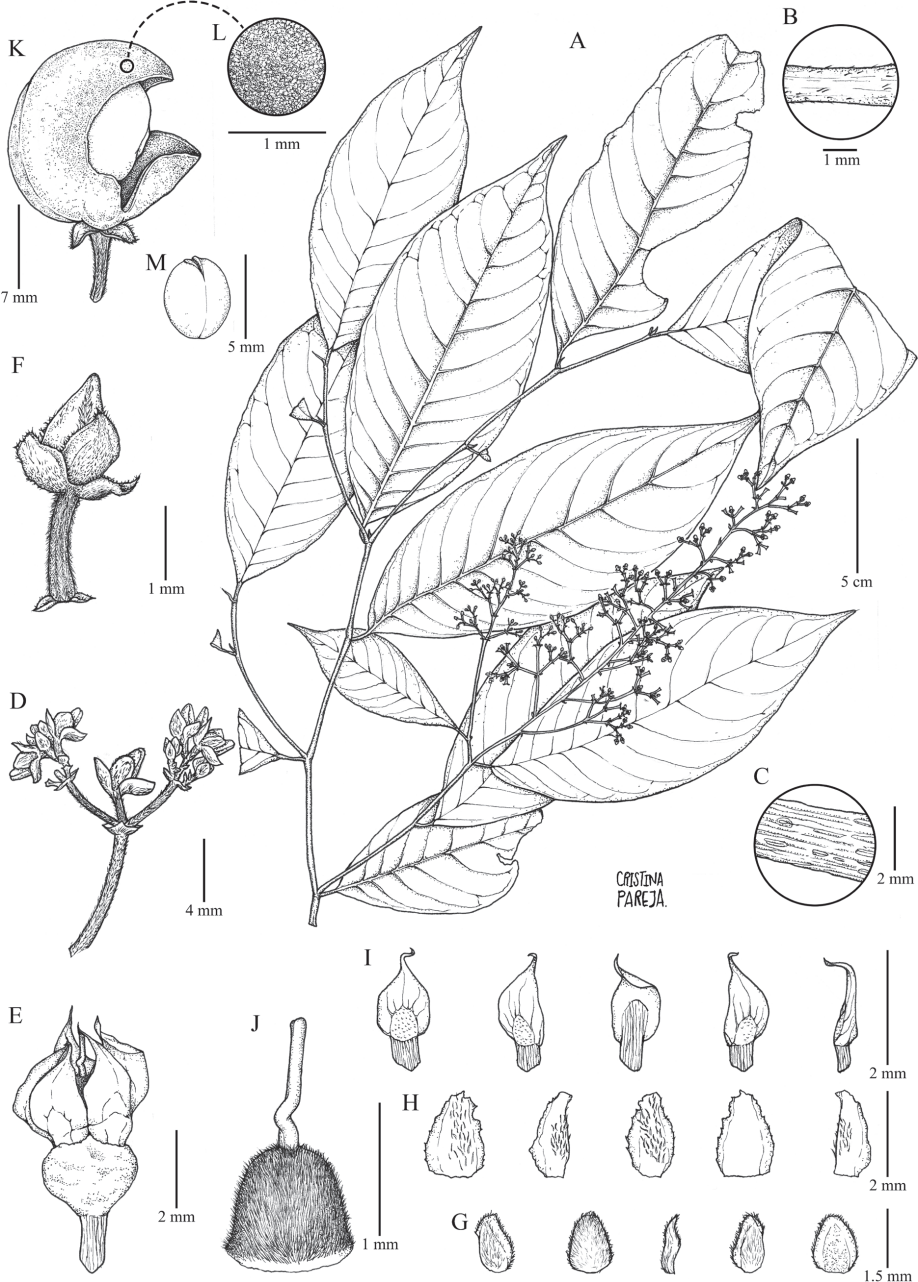
*Rinoreaidarragae* Hoyos-Gómez **A** habit **B** detail of pubescence on young twig **C** detail of lenticels on young twig **D** detail of cymule **E** detail of flower with fused filaments **F** flower in bud **G** sepals, abaxial and adaxial surfaces **H** petals, detail of abaxial and adaxial surface **I** stamens, detail of abaxial and adaxial surfaces **J** gynoecium **K** fruit **L** detail of fruit **M** seed. (**A–J**: *S. E. Hoyos-Gómez 1110* [HUA]; **K–M**: *J. Marín 12* [CR]).

Trees or treelets 6–16(20) m tall, terminal branchlets pubescent with appressed golden trichomes 0.1–0.3 mm long, glabrescent, with callose lenticels 0.3–0.5 mm long. Leaves alternate, petiolate; petiole 3–6 mm, pubescent with appressed golden trichomes 0.1–0.3 mm long; stipules deciduous, free, lanceolate, 2.5–6 × 1–1.5 mm, costa pubescent with appressed golden trichomes, 0.3–0.5 mm long, margin ciliolate, apex apiculate with mucron; lamina elliptic, 8–20 × 3–8 cm, foliaceous, adaxially glabrous, abaxially pubescent with appressed golden trichomes 0.1–0.3 mm long, glabrescent, semicraspedodromous, with 8 to 11 pairs of major secondary vein, secondary veins with equal spacing between them, base symmetrical, cuneate, margin subcrenate, apex acuminate, acumen 0.5–1.5 cm, mucronulate, leaf domatia absent. Inflorescence terminal, thyrsoid, solitary or often accompanied by one distinctly smaller lateral inflorescence, 5–15 × 3–5 cm, central axis brown, pubescent with appressed golden trichomes 0.1–0.3 mm long, cymules with 7 to 10(15) flowers; common peduncle 3–8 mm; pedicels 1–2 mm long, articulated near the base, pubescent with appressed golden trichomes 0.1–0.2 mm long; bracts persistent, triangular, 1–1.3 × 1–1.3 mm, with prominent costa, costa pubescent with appressed golden trichomes 0.1–0.3 mm long, margin ciliolate, apex apiculate, mucronulate; bracteoles persistent, triangular, 1 × 1 mm, costate, costa pubescent with appressed golden trichomes 0.1–0.3 mm long, margin ciliolate. Flowers subzygomorphic, 2.5–3 × 2.5–3 mm; sepals triangular, 1.4–1.6 × 1.3–1.5 mm, without veins or costa, pubescent with appressed golden trichomes 0.1–0.2 mm long, margin ciliolate, apex apiculate; petals white, elliptic, 1.8–2.3 × 1.3–1.5 mm, costa pubescent with golden appressed trichomes 0.1–0.3 mm long, margin ciliolate, apex acute; stamens 1.6–1.8 × 0.9–1.2 mm, filament connate at the base forming a glabrous staminal tube with glands covering the filament, filaments 0.2–0.3 mm, anthers ovoid, 0.5–0.7 × 0.4–0.6 mm, glabrous, apex rounded, connective 0.5–0.7 mm, dorsal anther connective scale lanceolate, 1.3–1.5 × 0.8–1 mm, cream, margin entire, apex acuminate; ovary globose, 0.7–0.9 × 0.7–0.9 mm, pubescent with appressed golden trichomes 0.2–0.4 mm long; style filiform, curved at the base, 1–1.2 mm long, glabrous, stigma acute. Fruit an asymmetrical subligneous capsule dehiscent along three sutures, ellipsoid, 1–1.3 × 0.8–1 cm, apex acuminate, densely tomentose with golden trichomes 0.1–0.2 mm long. Seeds one per valve, ovoid, 4.5–5.5 mm diam., glabrous, shiny brown when dry (Fig. 3).

##### Distribution and habitat.

**Figure 4.**
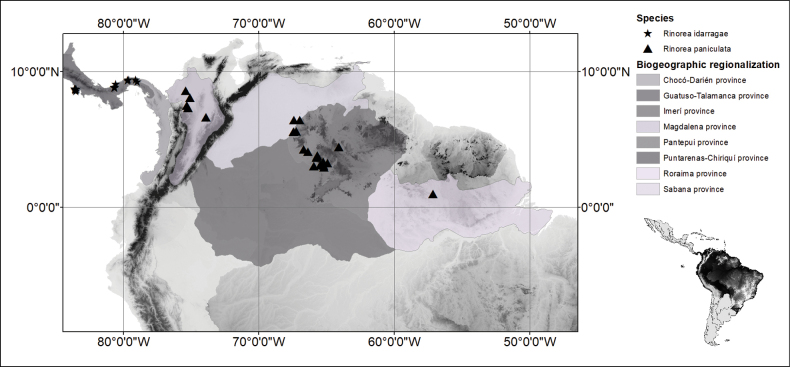
Distribution of *Rinoreaidarragae* (stars) and *R.paniculata* (triangles).

*Rinoreaidarragae* occurs in Costa Rica and Panama in the Punta Arenas-Chiriquí and Guatuso-Talamanca biogeographical provinces (sensu Morrone 2014), respectively (Fig. 4). The species occurs in primary tropical wet forests, often on ridges, at elevations of 50–300 m.

##### Etymology.

*Rinoreaidarragae* is named in honor of Alvaro Idárraga Piedrahíta, director of the JAUM herbarium of the Botanical Garden Joaquin Antonio Uribe in Medellín. Idárraga has made many contributions to the floristic knowledge of Antioquia Department in Colombia and the taxonomy of the Araliaceae.

##### Phenology.

The species flowers and fruits throughout the year.

##### Conservation status.

*Rinoreaidarragae* is known from 27 collections representing 21 occurrences. It has a geographic range in the form of an estimated EOO of 15,728 km^2^ and an AOO of 64 km^2^. While the species is well conserved in protected areas of the Osa Peninsula, Costa Rica, it is subject to mining and deforestation for timber extraction, agriculture, and grazing across the rest of its range in Panama. With regard to the most serious plausible threat of mining, the 21 occurrences represent 15 locations, and we project that ongoing mining and deforestation will lead to continuing decline in the area, extent and/or quality of habitat of the species. Nevertheless, the species does not meet the criteria for threatened status and is therefore preliminarily assessed as “Near Threatened” (NT) in accordance with the IUCN Red List Categories and Criteria (IUCN 2024).

##### Notes.

*Rinoreaidarragae* can be differentiated from *R.zygomorpha* H.E. Ballard & Wahlert by its 3 to 5× branched inflorescence (vs. 2 to 3× branched), cymules bearing 7 to 11 flowers (vs. 3–5), smaller sepals 1.4–1.6 × 1.3–1.5 mm and sepals lacking veins or costa (vs. larger sepals 1.9–2 × 1–1.5 mm and sepals 3- to 5-veined), smaller elliptic petals 1.8–2.3 × 1.3–1.5 mm (vs. larger broadly oblong petals 3.2–3.8 × 1.2–1.3 mm), smaller stamens 1.6–1.8 × 0.9–1.2 mm (vs. 3–3.5 mm), and smaller dorsal anther connective scale 1.3–1.5 × 0.8–1 mm with margin praemorse (vs. 2.7–3.2 × 0.9–1.1 mm with margin entire).

##### Additional specimens examined.

**Costa Rica**. **Prov. Puntarenas**: • Reserva Forestal Golfo Dulce Camino saliendo de Rancho Quemado a Quebrada Baneguas, 08°40'48"N, 83°31'12"W, 100–200 m, 31 Aug 1991 (fl), *R. A. Aguilar Fernández 291* (CR, MO); • Mpio. Golfito, Estación Los Patos, Sendero Sirena, 08°33'36"N, 83°30'36"W, 200 m, 2 Jun 1994 (fr), *R. A. Aguilar Fernández 3319* (CR, MO); • Osa, fila llegando a Rancho Quemado, 08°42'36"N, 83°33'36"W, 200 m, 1 Nov 1994 (fr), *R. A. Aguilar Fernández 3646* (CR, MO); • Reserve Forestal Golfo Dulce, Península de Osa, fila Casa Loma, Cerro Chocuaco, 08°41'39"N, 83°29'38"W, 200–300 m, 14 Apr 1998 (fr), *R. A. Aguilar Fernández 5405* (CR, MO); • Canton Osa, Distrito Sierpe, Reserva Forestal Golfo Dulce, Rancho Quemado, camino a Chiqueron en la parte mas alta de la fila al sur de Rancho Quemado, 630 m, 11 Nov 2001 (fl), *R. A. Aguilar Fernández 6709* (NY); • Distrito Sierpe, Reserva Forestal Golfo Dulce, Rincón, Banegas camino a Rancho Quemado en la primer cuesta, 192 m, 23 Jul 2009 (fl), *R. A. Aguilar Fernández 12214* (NY); • fila before Rancho Quemado, near Rincon, Osa Peninsula, 08°42'N, 83°33'W, 300 m, 11 Jan 1993 (fr), *A. H. Gentry et al. 78660* (CR, MO); • Rancho Quemado, Fila División, entre Banegas y Rancho Quemado, 08°40'48"N, 83°32'24"W, 300 m, 16 Jul 1991 (fl, fr), *J. Marín 12* (CR, MO); • Reserva Forestal Golfo Dulce Osa Península, Rancho Quemado; in forest on trail to Drake on ridge at N end of valley, on slope leading down to Guerra, 08°43'48"N, 83°36'00"W, 200 m, 2 May 1988 (fl, fr), *B. E. Hammel et al. 16785* (CM, CR, MO, NY); • Reserva Forestal Golfo Dulce Osa Península, Rancho Quemado, ca. 15 km W of Rincón; • at bottom of valley along Río Riyito near bridge and in forest along road on ridge above valley, 08°42'00"N, 83°33'00"W, 250–350 m, 31 May 1988 (fr), *B. H. Hammel et al. 16904* (MO); • along road between Rincón de Osa and Rancho Quemado, ca. 10 km W of main Rincón-Pto. Jimenez Road, 08°41'00"N, 83°32'30"W, 150–260 m, 3 Mar 1985 (fl), *T. B. Croat 59768* (CR, MO); • Rancho Quemado, finca de Jorge Cascante, 08°42'N, 83°34'W, 200 m, 5 Jul 1991 (fr), *F. J. Quesada 524* (MO). **Panama. Prov. Colón**: • Teck Cominco Petaquilla mining concession, collected near plot C003, 08°50'22"N, 80°38'51"W, 184 m, 18 Sep 2007 (fr), *G. D. McPherson 19677* (MO, US, NY); • Teck Cominco Mining Concession, Camp Colina, along road, 08°49'24"N, 80°39'45"W, 274 m, 27 Feb 2008 (fr), *M. C. Merello 3075* (MO); • Teck Cominco Petaquilla mining concession, along road, 08°49'18"N, 80°39'35"W–8°49'24"N, 80°39'48"W, 255–292 m, 28 Nov 2007 (fl), *H. van der Werff 22177* (MO, US); • San Juan del General, Conseción del Proyecto Mina de Cobre Panamá, Valle Grande, 08°49'18"N, 80°40'58"W, 280 m, 25 Jul 2014 (fl), *J. de García et al. 796* (MO, PMA); • Coclé del Norte, 09°04'30"N, 80°33'45"W, 100 m, 23 Aug 1978 (fl), *B. E. Hammel 4460* (MO); • Santa Rita Ridge, logging area 19 km from Transisthmian highway, 09°23'45"N, 79°39'15"W, 28 Jan 1968 (fr), *J. W. Dwyer 8556* (MO); • Santa Rita Ridge road, 4 mi from Transisthmian Highway to Agua Clara weather station, 09°21'N, 79°42'W, 500 m, 11 Dec 1973 (fl, fr), *A. H. Gentry 8830* (MBM, MO). **Prov. San Blás**: • Río Cagandí, hills W of river S of confluence with Río Titamibe, 09°24'00"N, 79°06'00"W–9°27'30"N, 79°07'00"W, 50–150 m, 27 Jan 1985 (fl), *G. C. de Nevers et al. 4666* (MO); • El Llano-Cartí road, north of Nusagandi, 2–6 miles, 09°15'N, 79°00'W, 250–300 m, 31 Mar 1988 (fl), *G. D. McPherson 12392* (MO, USCG); • Teck Cominco Petaquilla mining concession, 08°50'22"N, 80°38'51"W, 205 m, 18 Sep 2007 (fl), *G. D. McPherson 19683* (MO); • Teck Cominco Petaquilla mining concession, 08°49'28"N, 80°39'29"W, 190 m, 21 Sep 2007 (fl, fr), *G. D. McPherson 19772* (MO); • *ibid*., 08°49'18"N, 80°39'35"W, 255 m, 28 Nov 2007 (fl, fr), *G. D. McPherson 19850* (MO, US); • *ibid*., (fr), *G. D. McPherson 19856* (MO); • Teck Cominco Mining Concession, Colina Camp, along road, 08°49'23"N, 80°39'32"W, 101 m, 26 Feb 2008 (fl), *M. C. Merello et al. 3058* (MO).

#### ﻿3. Rinorea pabongonzaleziorum

Taxon classificationPlantaeMalpighialesViolaceae

Hoyos-Gómez sp. nov.

6B7C8E68-B211-5562-ABEF-5CFD09E912E1

urn:lsid:ipni.org:names:77362481-1

##### Type.

**Colombia**. Dept. Vaupés: • Mpio. Mitú, above Mitú, bank of río Vaupés, 200 m, 24 Mar 1970, *D. D. Soejarto 2315* (holotype: HUA [acc. no. 3197; barcode HUA–0019764]!; isotype: F [acc. no. 1835143]!).

##### Diagnosis.

*Rinoreapabongonzaleziorum* resembles *R.macrocarpa* (Mart. ex Eichler) Kuntze by the opposite leaves lacking domatia, racemose inflorescence, praemorse margin of the dorsal anther connective scale, and filaments connate at the base forming a staminal tube, but it differs by the obovate lamina (vs. elliptic lamina in *R.macrocarpa*), 4 to 6 major secondary vein pairs (vs. 7 to 9), two glabrous seeds per valve (vs. three), smaller fruits 2.5–3.2 × 2–2.5 cm (vs. 3.5–5.8 × 2.5–3 cm), and smaller seeds 5–7 mm diam. (vs. 7.5–9 mm diam.).

##### Description.

**Figure 5.**
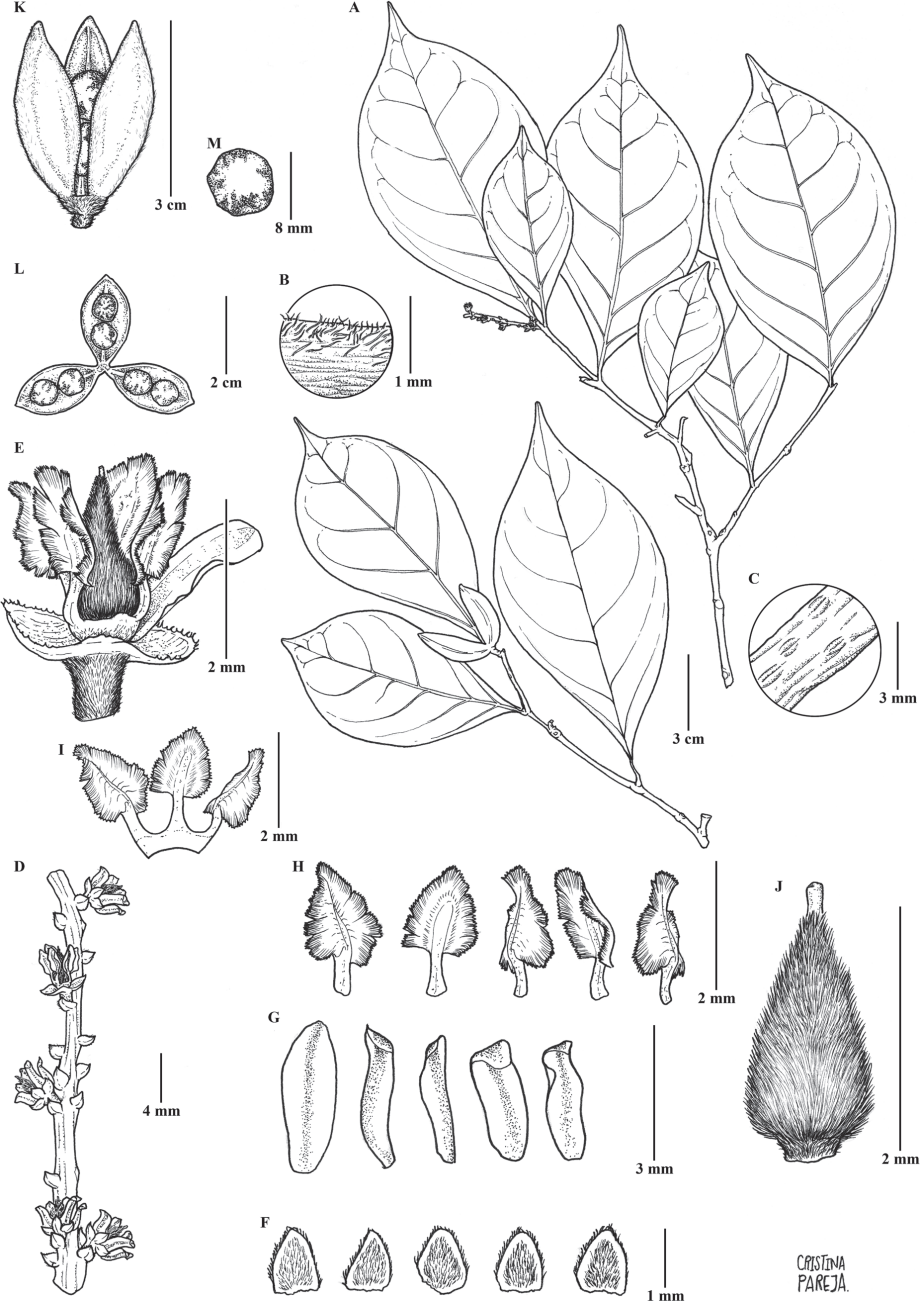
*Rinoreapabongonzaleziorum* Hoyos-Gómez **A** habit **B** detail of pubescence on young twig **C** detail of lenticels on young twig **D** inflorescence **E** flower **F** sepals abaxial surface **G** petals, detail of abaxial and adaxial surface **H** stamens, detail of abaxial and adaxial surface **I** androecium, detail of fused stamens forming a staminal tube **J** gynoecium **K** fruit **L** detail of open fruit showing two seeds per valve **M** seed (**A–M**: *D. D. Soejarto 2315* [HUA]).

Treelets 3–5 m tall, terminal branchlets pubescent with appressed ferruginous trichomes 0.1–0.3 mm long, callose lenticels 0.5–0.6 mm long, brownish when dry. Leaves opposite, petiolate; petiole 3–6.5 mm, pubescent with appressed ferruginous trichomes 0.1–0.3 mm long; stipules deciduous, free, lanceolate, 2–4 × 1–1.5 mm long, densely pubescent with appressed golden trichomes, 0.2–0.3 mm long; lamina obovate, 6–15 × 4–8 cm, foliaceous, adaxially glabrous, abaxially pubescent on midvein, secondary and tertiary veins with appressed scattered golden trichomes 0.3–0.5 mm long, veins pinnate, semicraspedodromous, with 4 to 6 pairs of major secondary vein, secondary veins with unequal spacing between them, percurrent, base symmetrical, cuneate, margin subcrenate to subentire, apex acuminate, acumen 1–1.8 cm, mucronulate, leaf domatia absent. Inflorescence terminal, racemose, solitary, 3–4 × 0.8–1.5 cm, central axis brown, densely pubescent with erect ferruginous trichomes 0.2–0.3 mm long; pedicels 0.5–0.9 mm, pubescent with erect ferruginous trichomes 0.2–0.3 mm long, articulated at the base; bracts persistent, triangular, 1–1.2 × 1–1.2 mm long, costa pubescent with appressed golden ferruginous trichomes 0.2–0.3 mm long, margin ciliolate, mucronulate; bracteoles persistent, triangular, 0.5–0.6 × 0.5–0.6 mm, opposite, costate, pubescent with appressed ferruginous trichomes 0.2–0.3 mm long, margin ciliolate, apex acuminate, mucronulate. Flowers 3–4 × 3–4 mm, subzygomorphic; sepals triangular, 1–1.2 × 0.8–0.9 mm, densely pubescent with appressed golden trichomes 0.2–0.3 mm long, margin ciliolate, apex apiculate; petals white, lanceolate, 3–3.5 × 1.2–1.5 mm, costa pubescent with scattered ferruginous trichomes 0.3–0.4 mm long, glabrescent, margin entire, apex reflexed; stamens 2.3–2.6 × 1–1.3 mm, filaments connate at the base forming a glabrous staminal tube with glands covering the filament, tube 0.5–0.6 mm tall, anthers elliptic to ovoid, 0.8–0.9 mm, glabrous, dorsal anther connective scale lanceolate, 1.4–1.7 × 1.3–1.5 mm, margin strongly fimbriate, cream-colored; ovary pyriform, 2–2.2 × 1.3–1.5 mm, densely pubescent with appressed golden trichomes 0.3–0.5 mm long; style filiform, erect, 0.4–0.6 mm long, glabrous, stigma acute. Fruit a symmetrical subligneous capsule dehiscent along three sutures, ellipsoid, 2.5–3.2 × 2–2.5 cm, apex acuminate, pubescent with golden uncinate trichomes 0.1–0.2 mm long. Seeds two per valve, globose, 5–7(8) mm diam., glabrous, with maculae, brown when dry (Fig. 5).

##### Distribution and ecology.

*Rinoreapabongonzaleziorum* is endemic to the border region between Colombia and Venezuela, in the Imerí and Pantepui biogeographical provinces (sensu Morrone 2014; Fig. 2). It occurs in primary and secondary tropical wet forest, often on hill slopes, at elevations from 140 to 300 m.

##### Etymology.

*Rinoreapabongonzaleziorum* is named in honor of Professors Dr. Natalia Pabón M. and Dr. Favio González G., who have made significant contributions to the knowledge of the flora of Colombia. Professor Natalia Pabon M. is a specialist in floral evolution and development at Universidad de Antioquia, Medellín, and Professor Favio González G., at the Universidad Nacional de Colombia, Bogotá, specialized in the taxonomy of South American Aristolochiaceae and Campanulaceae.

##### Phenology.

The species was recorded in flower in February and March and September through December. It was in fruit in February, March, and December.

##### Conservation status.

*Rinoreapabongonzaleziorum* is known from 14 collections representing nine occurrences. It has a geographic range in the form of an estimated EOO of 211,657 km^2^ and an AOO of 32 km^2^. The species occurs in Serranía La Neblina National Park in Venezuela, but across the rest of its range, it is subject to deforestation for timber extraction, agriculture, and grazing. We project that ongoing deforestation will lead to continuing decline in the area, extent and/or quality of habitat of the species. With regard to the most serious plausible threat of deforestation, the nine occurrences represent six locations. Therefore, *R.pabongonzaleziorum* is preliminarily assessed as “Vulnerable” [VU B2ab(iii)] in accordance with the IUCN Red List Categories and Criteria (IUCN 2024).

##### Additional specimens examined.

**Colombia. Dept. Vaupés**: • Mpio. Taraira, comunidad Jotabeyá, caño Jotabeyá, mas adentro del río Apaporis, varador “Chorro de Yarumo,” 0°35'S, 70°11'W, 150–250 m, 27 Mar 2009 (fr), *J. C. Betancur B. 13854* (COAH, COL, HUA); • Mpio. Mitú, km 11.2 carretera Mitú–Montfort, Sep 1993 (fl), *X. Martínez et al. 1322* (HUA); • Mpio. Mitú, Caño Cucura, 01°09'39"N, 70°08'49"W, 147 m, 7 Dec 2021 (fl, fr), *S. E. Hoyos-Gómez 4850* (HUA, JAUM, COAH, MO, US, NY); • Carretera Mitú-Tayazu, 01°05'09"N, 70°03'22"W, 180 m, 22 Ago 2024 (fr), *S. E. Hoyos-Gómez 5792* (HUA, JAUM, COAH); • Río Guainía, Puerto Colombia (opposite Venezuelan town of Maroa) and vicinity, 249–254 m, 31 Oct–2 Nov 1952 (fl), *R. E. Schultes 17932* (US); • *ibid.*, *R. E. Schultes 17933* (US, GH). **Venezuela**. **Amazonas State**: • Dept. Río Negro, Neblina Base Camp, Río Mawarinuma, 0°50'N, 66°10'W, 140 m, 25 Nov 1984 (fl), *B. B. Boom 5132* (MO, NY, GH); • *ibid*., 2 Feb 1984 (fl, fr), *B. B. Boom 5536* (INPA, K, MO, US); • Río Negro, 1.5 km south of Cerro de La Neblina Base Camp, which is on Río Mawarinuma, 0°50'N, 66°10'W, 140 m, 12 Mar 1984 (fl, fr), *R. L. Liesner 16554* (BHO, MO, VEN); • *ibid*., 27 Nov 1984 (fr), *R. L. Liesner 17352* (F, MO, US); • Cerro Neblina base camp along Río Mawarinuma, ca. 0°50'N, 66°10'W; 140 m, 2 Dec 1984 (fl), *T. B. Croat 59562* (F, MO, NY, US); • Dpto. Atures, 125 km de la boca (delta) del Guayapo en Sipapo, 04°22'N, 67°06'W, 130 m, May 1989 (fr), *E. Foldats et al. 9187* (MO, NY); • Mpio. Foraneo Aripao, margen derecha del caño Minchaquene (Hormiga), tributario del Alto Caura, entre Araguaña y Campamento, 7°20'N, 65°10'W, 300 m, 2–5 May 1988 (fr), *G. A. Aymard C. et al. 6810* (MO).

### ﻿New combinations

#### ﻿4. Rinorea fernandeziana

Taxon classificationPlantaeMalpighialesViolaceae

(Hekking) Hoyos-Gómez, comb. et stat. nov.

E0501524-483E-5F23-8EE1-476E192FD5F5

urn:lsid:ipni.org:names:77362482-1

≡ Rinorealindenianavar.fernandeziana Hekking, Phytologia 43: 482. 1979. Type. Colombia. Prov. Chocó, Coredó, along the Pacific coast, 16 Jun 1950, 0 m, *A. Fernández-Pérez 365* (holotype: COL [acc. no. 34415]!; isotypes: NY [acc. 00073974]!; US [cat. no. 1998021; barcode US-00114491]!).

##### Description.

Trees or treelets 3–10 m tall, terminal branchlets pubescent with dispersed ferruginous trichomes 0.1–0.2 mm long, glabrescent, with white lenticels 0.4–0.6 mm. Leaves opposite, petiolate; petiole 4–7(10) mm long, pubescent with erect ferruginous trichomes 0.1–0.2 mm long; stipules deciduous, free, lanceolate, 1.5–3 × 0.5–1.5 mm, pubescent with appressed ferruginous trichomes, 0.2–0.3 mm long; lamina elliptic, 8–16 × 3–7 cm, foliaceous, glabrous, veins pinnate, brochidodromous, with 6 to 8 major secondary vein pairs, secondary veins with unequal spacing between them, percurrent, base symmetrical, cuneate, margin subcrenate to subentire, apex acuminate, acumen 4–8 mm, mucronulate, leaf domatia absent. Inflorescence terminal, racemose, solitary, (3)5–7(8) × 0.5–0.9 cm, central axis pubescent with erect ferruginous trichomes 0.1–0.2 mm long; pedicels 1–1.5 mm long, articulated near the middle; bracts persistent, triangular, 0.8–1 × 0.5–0.8 mm long, costa moderately pubescent with golden ferruginous trichomes 0.2–0.3 mm long, margin ciliolate, apex acuminate, mucronulate, bracteoles deciduous, triangular, 0.5–0.8 mm long, opposite, costate, pubescent with ferruginous trichomes 0.2–0.3 mm long, margin ciliolate, apex acuminate, mucronulate. Flowers 4–5 × 4–5 mm, subzygomorphic; sepals ovate, 1–2 × 1–2 mm, with 3–5 longitudinal veins, pubescent with ferruginous trichomes 0.2–0.3 mm long, glabrescent, margin ciliolate, apex apiculate; petals lanceolate, 2.5–3.5 × 1–2 mm, glabrous, entire margin, rounded apex, white when fresh, brownish when dry; stamens 2–3 mm long, filaments free, glabrous, 0.6–0.8 mm long, dorsal gland free, 0.6–0.8 mm long, glabrous, anthers ovoid, 1–1.3 × 0.3–0.4 mm, glabrous, dorsal anther connective scale lanceolate, 1–1.5 × 0.4–0.8 mm, margin fimbriate; ovary subglobose, 0.7–1.3 × 0.5–1.2 mm, pubescent with appressed ferruginous trichomes 0.2–0.3 mm long; style filiform, erect, 0.8–1.3 mm long, glabrous, enlarged distally, stigma acute. Fruit a symmetrical subligneous capsule dehiscent along three sutures, ellipsoid, 1.5–1.8 × 1–1.5 cm, apex acuminate, pubescent with dimorphic ferruginous trichomes, small abundant appressed trichomes 0.1–0.2 mm long and scattered longer erect trichomes 0.4–0.5 mm long. Seeds one per valve, globose, 6–7(8) mm diam., pubescent with ferruginous erect trichomes 0.3–0.5 mm long, with maculae, brown when dry.

##### Distribution and habitat.

**Figure 6.**
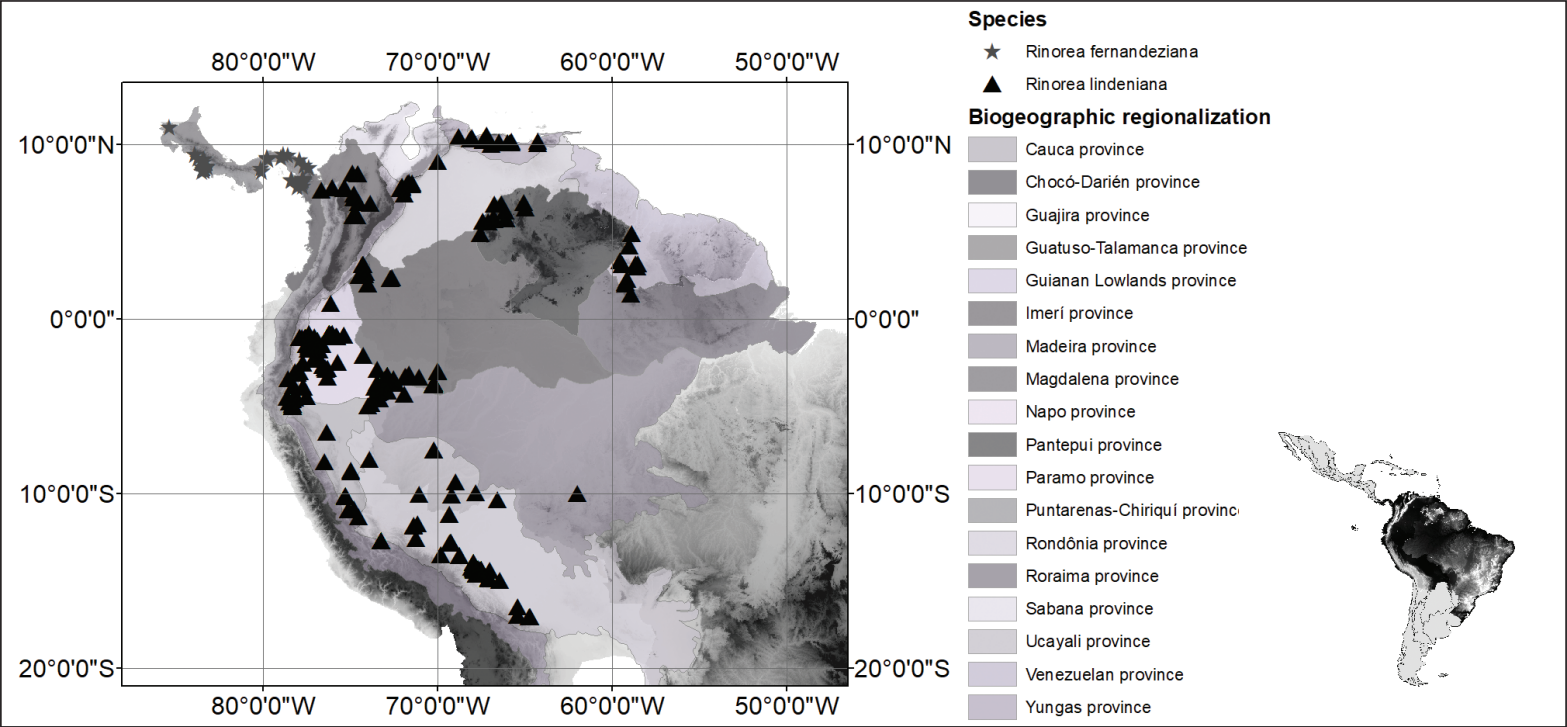
Distribution of *Rinoreafernandeziana* (stars), and *R.lindeniana* (triangles).

*Rinoreafernandeziana* occurs in the Pacific Coast regions of Costa Rica, Panama, and Colombia that correspond to the Guatuso–Talamanca biogeographical province (sensu Morrone 2014; Fig. 6). It grows in lowland and premontane wet forests, often on ridges and near rivers, at elevations of 20 to 1030 m.

##### Phenology.

The species flowers and fruits throughout the year.

##### Notes.

Hekking (1979) differentiated Rinorealindenianavar.fernandeziana from the nominate species based on the symmetrical and decurrent leaf lamina base (vs. asymmetrical and subauriculate in var. lindeniana), the shorter racemose inflorescences (vs. longer thyrsoid inflorescences), larger sepals 1.5–2 × 1.5–2 mm (vs. 0.75–1.5 × 0.75–1.5), and style subclavate (vs. sigmoid). The two taxa have different geographic distributions, with *R.fernandeziana* occurring in Costa Rica and Panama and the northern portion of the Darien region and *R.lindeniana* occurring in the inter-Andean valleys of Colombia south to the lowland areas of Colombia, Venezuela, Trinidad, Brazil, Peru, and Ecuador.

##### Additional specimens examined.

**Colombia**.• **Dept. Chocó**: • Mpio. de Acandí, corregimiento de Capurganá, bahía el Aguacate, Serranía del Darién, 08°37'N, 77°18'W, 250–350 m, 10 Jan 2007 (fr), *J. C. Betancur B. & S. E. Hoyos-Gómez 12477* (HUA, COL); • Reserva Natural el Aguacate, 08°36'58.6"N, 77°19'41.6"W, 200–250 m, 23 Jul 2008 (fr), *S. E. Hoyos-Gómez et al. 796* (HUA, COL); • Vereda el Brillante, la Paloma camino a los pozos y alrededores, 08°34'59.4"N, 77°23'09.9"W, 80–120 m, 28 Jan 2018 (fr), *S. E. Hoyos-Gómez et al. 3378* (HUA); • SW Ridge leading to alturas de Nique, Colombia and Panama border, 07°35'30"N, 77°44'00"W, 800–900 m, 28 Dec 1980 (fr), *R. L. Hartman 12326* (MO); • Hydro Camp 14, Río Salaqui, 6 days upstream from Río Sucio, 200 m, 23 May 1967 (fl), *J. A. Duke 11374* (MO). **Costa Rica**. **Prov. Guanacaste**: • P.N. Guanacaste, estación Pitilla, La Cruz, 10°59'26"N, 85°25'40"W, 700 m, 7 Nov 1990 (fl), *C. Moraga et al. 215* (MO). **Prov. Puntarenas**: • Península de Osa, estación El Tigre, cabecera del Río Agujas, finca Azofeifa, 08°31'48"N, 83°27'36"W, 700 m, 19 Nov 1993 (fl, fr), *R. A. Aguilar Fernández 2659* (MO); • *ibid*., 08°31'N, 83°25'W, 200 m, 5 May 1994 (fl), *R. A. Aguilar Fernández 3218* (MO); • P.N. Corcovado, Península de Osa, estación El Tigre, cabecera Río Agujas, finca Azofeifa, 08°31'N, 83°25'W, 200 m, 8 May 1994 (fl), *R. A. Aguilar Fernández et al. 3247* (MO); • Cuenca, Terraba-Sierpe, Quebrada Guerra, Reserva Montana Tigre, 09°09'N, 83°26'W, 400 m, 10 Feb. 2000 (fr), *R. A. Aguilar Fernández 5933* (MO); • Cantón de Golfito P.N. Corcovado, Península De Osa Bonanza, 08°31'30"N, 83°25'40"W, 5 Mar 1997 (fr), *A. Azofeifa 259* (MO); • *ibid*., 08°32'11"N, 83°25'31"W, 21 Nov 1996 (fl, fr), *A. Azofeifa 227* (MO); • Uvita, San Josecito, faldas de la Fila Alivio, finca Oro Verde, 09°12'30"N, 83°45'47"W, 400–500 m, 2 Feb 2000 (fr), *M. A. Blanco et al. 1379* (MO); • hills above Palmar Norte, 100–200 m, 20 May 1976 (fl), *T. B. Croat 35130* (MO); • R.F. Golfo Dulce, Serranías de Golfito, estación Río Bonito, Sendero a San Josecito, 08°41'25"N, 83°14'35"W, 250–300 m, 23 May 1996 (fl), *E. Fletes 316* (MO); • Fila before Rancho Quemada, near Rincón, Osa Península, 08°42'N, 83°33'W, 300 m, 11 Jan 1993 (st), *A. H. Gentry et al. 78675* (MO); • finca El Edén, km 183, Route 2, ca. 400 m E of Santa Marta, 09°09'29"N, 83°23'37"W, 400 m, 6 Sept 1984 (fl, fr), *L. D. Gómez 22958* (MO); • P.N. Corcovado, Península de Osa, trayecto entre la Quebrada La Bonanza y el Cerro Rincón, por la Fila Matajambre, 300–745 m, 7 May 1994 (fl), *J. A. González et al. 255* (MO); • Ridge between Quebrada Aparicio and Quebrada Aguabena, S and W of Rincón de Osa, 08°42.5'N, 83°30'W 40–200 m, 25 May 1986 (fl), *M. H. Grayum et al. 7554* (MO); • Reserva Forestal Golfo Dulce Osa Península, trocha de La Tarde Rd. 10 km SW of La Palma, S of Rincón de Osa, ridge E of the Río Rincón valley, 08°37'N, 83°28'W, 150–200 m, 28 Apr 1988 (fr), *B. E. Hammel et al. 16745* (F, MO); • P.N. Corcovado, El Tigre/Cerro Mueller, 08°27'N, 83°33'W–8°30'N, 83°38'W, 150–650 m, 22 May 1988 (fl), *C. Kernan et al. 519* (MO); • R.F. Golfo Dulce, Península de Osa, Río Agujas, estación Agujas, Sendero Zamia, 08°32'12"N, 83°25'32"W, 300 m, 9 Oct 1997 (fl, fr), *M. Lobo 120* (MO); • Golfito P.N. Corcovado, Península de Osa, estación Agujas, Sendero Zamia, 08°31'52"N, 83°26'00"W, 300 m, 03 Nov 1999 (fl), *E. Mora et al. 590* (MO); • P.N. Corcovado, Valle de Coto Colorado, Punta Estrella y Punta Bejuco, 08°46'00"N, 83°15'00"W, 100 m, 8 Nov 1993 (fl), *F. J. Quesada 802* (MO). • Prov. San José: Pérez Zeledón, Río Nuevo, Viento Fresco, 09°24'46"N, 83°56'12"W, 609 m, 8 Feb 2001 (fr), *S. Lobo et al. 300* (MO); • Cuenca del Savegre, Río Nuevo, 1.6 km SE de Viento Fresco, 09°24'46"N, 83°56'12"W, 600 m, 08 Feb 2001 (fr), *A. Rodriguez et al. 7021* (MO). **Panama** • Without precise locality: *J. A. Duke 6552* (MO); *J. A. Duke 15685* (MO). **Prov. Darién**: • Cerro Pirre, 07°52'N, 77°44'W, 11 Apr 1967 (fr), *N. Bristan 578* (MO); • Middle slopes on W side of Cerro Pirre, 07°57'N, 77°46'W, 550–760 m, 28 Jun 1988 (fl, fr), *T. B. Croat 68860* (MO); • Pipeline Road, N of Gamboa, upstream of the tenth bridge (Río Guacharo), beyond the big waterfall, 09°10'N, 079°45'W, 100 m, 04 Aug 1984 (fl, fr), *G. C. de Nevers 3619* (MO); • trail from mouth of Río Irgandí to a tributary of Río Cartí Senni, 09°25'N, 78°51'W, 20 Dec 1985 (fr), *G. C. de Nevers 6578* (MO); • Coclé, near El Valle, 08°36'N, 80°08'W, 4 Aug 1963 (fl), *J. A. Duke & H. W. Mussell 6592* (MO); • area around Rancho Frío, half-way up slope of Cerro Pirre from Piji Vasal, 08°01'N, 77°44'W, 12 Nov 1977 (fr), *J. P. Folsom 6238* (MO); • along bank of Río Aqua Salud between Frijoles and Pipeline Road, 100 m, 21 Oct 1970 (fl, fr), *R. B. Foster et al. 1950* (MO); • Río Pirre, near Dos Bocas, 08°01'N, 77°44'W, 04 Aug 1975 (fl), *R. B. Foster et al. 2831* (MO); • Pipeline Road, 4 miles N of Gamboa, 50–100 m, 21 Dec 1971 (st), *A. H. Gentry et al. 3244* (MO); • *ibid*., 6–8 mi N of Gamboa, 100 m, 29 Dec 1971 (st), *A. H. Gentry et al. 3369* (MO); • Pipeline road, 50–120 m, 11 Mar 1983 (fr), *A. H. Gentry et al. 41127* (MO); • Cerro Sapo, 07°58'N, 78°22'W, 762 m, 1 Feb 1978 (fr), *B. E. Hammel 1192* (MO); • *ibid*., 3 Feb 1978 (fl), *B. E. Hammel* 1318 (MO); • South of Garachiné, near Pacific coast, above Casa Vieja, along boundary trail of P.N. Darién, W flank Serranía Sapo, 07°56'N, 78°24'W, 50–150 m, 21 May 1991 (fl), *N. C. Hensold 1073* (MO); • P.N. Darién, Serranía de Cerro Sapo, por la trocha limítrofe del PND entre Casa Vieja y Cerro Sapo, 07°58'N, 78°23'W, 20–400 m, 24 Nov 1990 (fr), *H. Herrera et al. 745* (MO); • *ibid.*, 07°56'N, 78°23'W–7°58'N, 78°24'W, 150–300 m, 22 May 1991 (fl), *H. Herrera et al. 978* (MO); • vecindad de Yannuadi, Isla de Narganá, a 5 km de la costa, 09°22'N, 78°35'W, 50–100 m, 23 Oct 1992 (fr), *H. Herrera et al. 1232* (MO); • Pipeline Road, Agua Salud, 1 Nov 1972 (fl, fr), *H. Kennedy et al. 1886* (MO); • Cuasí-Cana Trail on Cerro Campamento east of Tres Bocas, headwater of Río Cuasi, 07°46'N, 77°47'W, 450 m, 29 Apr 1968 (fl), *J. H. Kirkbride, Jr.1255* (BHO, MO); • near Puerto Obaldía, W of village, on foot-trail to La Bonga, 08°40'N, 77°25'W, 50–140 m, 24 Mar 1985 (fr), *G. D. McPherson 6966* (MO); • S of El Real on trail up Cerro Pirre, 08°00'N, 77°45'W, 550–1030 m, 29 Mar 1985 (fr), *G. D. McPherson 7049* (MO); • *ibid*., (fl), *G. D. McPherson 7050* (MO); • on El Llano-Carti road; • near Nusigandí, along Wedar trail, 09°15'N, 79°00'W, 250 m, 31 Oct 1992 (fr), *G. D. McPherson 15996* (MO); • north slopes of Cerro Pirre, 08°00'N, 77°42'W, 300–700 m, 8 Apr 1975 (fr), *S. A. Mori et al. 5506* (MO); • *ibid*., (st), *S. A. Mori et al. 5513* (MO); • along Río Mendoza and small tributary, 0.5–1 km upstream from Pipeline Road bridge, 8 km NW of Gamboa, 100 m, 1 Nov 1973 (fl), *M. H. Nee 7734* (MO); • canal area, along Río Mendoza 0.5 km downstream from Pipeline Road bridge, 8 km NW of Gamboa, 09°09'N, 79°44'W, 90 m, 9 Feb 1974 (st), *M. H. Nee 9570* (MO); • Sarsib Yala, tierra firme frente a Isla Ustupu, por el camino del acueducto, 09°07'N, 77°55'W, 0–100 m, 15 Feb 1988 (fr), *R. Paredes 404* (MO); • Aila Terrain (Río Acla), 08°48'30"N, 77°40'30"W, 25–100 m, 16 Jan 1979 (fr), *A. M. Sugden 353* (MO); • San Blás, 08°48'30"N, 77°40'30"W, 25–100 m, 16 Mar 1979 (fl), *A. M. Sugden 547B* (MO); • 10 km NE of Jaqué slopes of Río Tabuelitas above Birogueirá, Indian village on Río Jaqué below mouth of Río Pavarandó, 07°33'N, 78°05'W, 400 ft, 30 Jan 1981 (fr), *K. J. Sytsma et al. 3330* (MO).

### ﻿New synonymies

#### ﻿5. Rinorea laurifolia

Taxon classificationPlantaeMalpighialesViolaceae

L.B. Sm. & Á. Fernández, Caldasia 6: 90. 1954.

38ECC85F-63BC-5B61-8ED4-5F8C849E562D

= Rinoreacordata L.B. Sm. & Á. Fernández, Caldasia 6: 90. 1954, syn. nov. Type. Colombia. Dept. Santander: camp on Margarita Creek, vicinity of Barranca Bermeja, between Sogamoso and Colorado Rivers, 100–500 m, 10 Oct 1934, *O. L. Haught 1388* (holotype: US [barcode 00114471; cat. no. 1592066]!; isotype COL [barcode COL000002869]!).

##### Type.

Colombia • Dept. Santander: vicinity of Puerto Berrio, between Carare and Magdalena Rivers, 100–700 m, Jul 1936, *O. L. Haught 1908* (holotype: COL [barcode COL000002872]!; isotype: US [barcode 00114483; cat. no. 1661881]!).

##### Notes.

The type localities of *Rinorealaurifolia* and *R.cordata* are separated by less than 40 km in the area between Barrancabermeja and Puerto Berrío on the Magdalena River. Both of the localities are also similar in terms of vegetation type and climate. The two species were described from fragmentary herbarium material, yet after examination of the types and several additional specimens, the nearly 20 qualitative and quantitative morphological characters we measured were found to overlap considerably. *Rinoreacordata* clearly fit within the circumscription of *R.laurifolia*, and we were unable to characterize any consistent differences that would allow us to differentiate or maintain the two as separate taxa.

#### ﻿6. Rinorea ulmifolia

Taxon classificationPlantaeMalpighialesViolaceae

(Kunth) Kuntze, Revis. Gen. Pl. 1: 42. 1891.

7D1D4BF6-E168-5FDC-96CD-4313B37B8D30

≡ Conohoriaulmifolia Kunth, Nov. Gen. Sp. (quarto ed.) 5: 387. 1823.
≡ Alsodeiaulmifolia (Kunth) Spreng., Syst. Veg. 1: 807. 1825 [1824].
= Rinoreahymenosepala S. F. Blake, Contrib. U.S. Natl. Herb. 20: 504. 1924, syn. nov. Type: Colombia. Antioquia: Malena, 12 Jan 1918, 150–170 m, *F. W. Pennell 3783* (holotype: NY [barcode 00095077]!).

##### Type.

Colombia • Antioquia: Rio Magdalena, propé Bartholomé, May 1801, *A. J. A. Bonpland 1608* (holotype: P [barcode P02141374]!).

##### Notes.

The type localities of *Rinoreaulmifolia* and *R.hymenosepala* are in the immediate vicinity of Puerto Berrío on the Magdalena River and are similar in terms of vegetation type and climate. In the description of *R.hymenosepala*, Blake (1924) notes that the new species most closely resembles *R.ulmifolia*, however, when citing the type of *R.ulmifolia*, he states that he did not actually examine it. One character Blake used to differentiate the two species was the margin of the dorsal anther connective scale: erose in *R.hymenosepala* and entire in *R.ulmifolia*. In reality, examination of the types and associated specimens all show that the anther scale margin is erose near the base. Another character used to differentiate the two taxa was the phyllotaxy: *Rinoreahymenosepala* appears to have alternate leaf arrangement on proximal portions of the stem and opposite arrangement on distal portions, whereas *R.ulmifolia* is consistently opposite. Field observations of whole plant architecture of species in Rinoreasect.Pubiflora do indeed show that leaves growing on first-year or very young stems are spirally arranged, but when the plant matures, the phyllotaxy becomes opposite. The type specimen of *R.hymenosepala* was taken from a very young plant and shows this pattern of phyllotaxy, which has been observed in the field in several species in R.sect.Pubiflora. After examining many other morphological characters, we found no differences that would allow us to maintain *Rinoreahymenosepala* as distinct from *R.ulmifolia*. In fact, the type specimen of *R.hymenosepala* fits neatly within the range of morphological variation of *R.ulmifolia*.

#### ﻿7. Rinorea squamata

Taxon classificationPlantaeMalpighialesViolaceae

S.F. Blake, Contrib. U.S. Natl. Herb. 20: 516. 1924.

7F7BB0CE-5181-5F56-8AC3-4D1FFEEC89AB

= Rinoreapubipes S. F. Blake, Contrib. U.S. Natl. Herb. 20: 515. 1924, syn. nov. Type: Costa Rica. Limón: Zent Farms, ca. 30 km W of Puerto Limon, s.d., *H. F. Pittier s.n.* (holotype: US [cat. no. 578517, barcode 00114502]!; isotype F [acc. 1709848]!).
= Rinoreaantioquiensis L.B. Sm. & Á. Fernández, Caldasia 6: 105. 1954, syn. nov. Type: Colombia. Antioquia: SE of Chigorodo, 40 km S of Turbo, 50 m, 15 Apr 1945, *O. L. Haught 4566* (holotype: US [cat. no. 1709146, barcode 00114466]!; isotypes: COL [acc. no. 30955; barcode COL000002868]!; K [barcode K000327516]!; NY [barcode 00095074]!).

##### Type.

Panama • Canal zone, near Gatun, 10 Feb 1911, *E. A. Goldman 1864* (holotype: US [cat. no. 690300; barcode 00114504]!).

##### Notes.

*Rinoreasquamata* and *Rinoreapubipes* were both described by Blake (1924), with the latter synonymized under *R.squamata* by Hekking (1988). Later, Smith and Fernández (1954) described *Rinoreaantioquiensis* from a single collection. After careful study of vegetative and reproductive morphology of the type specimens, no distinguishing characters could be found to separate the three taxa. *Rinoreasquamata* is morphologically uniform across its wide range, from Central America (Honduras, Nicaragua, Costa Rica, Panama) to northern Colombia.

### ﻿Updated descriptions

#### ﻿8. Rinorea deflexa

Taxon classificationPlantaeMalpighialesViolaceae

(Benth.) S.F. Blake, Contrib. U.S. Natl. Herb. 20: 513. 1924.

C9A375D2-01D0-5399-B150-FAB1EAC1D600

≡ Alsodeiadeflexa Benth., Bot. Voy. Sulphur: 67. 1844.

##### Type.

Ecuador. Esmeraldas: • Atacames, s.d., *G. W. Barclay s.n.* (holotype: K [barcode K000327511]!; isotypes: K [barcode K000327512; specimen in same type folder as K000327511]!; US [cat. no. 1056900; barcode 00114474]!).

##### Description.

**Figure 7.**
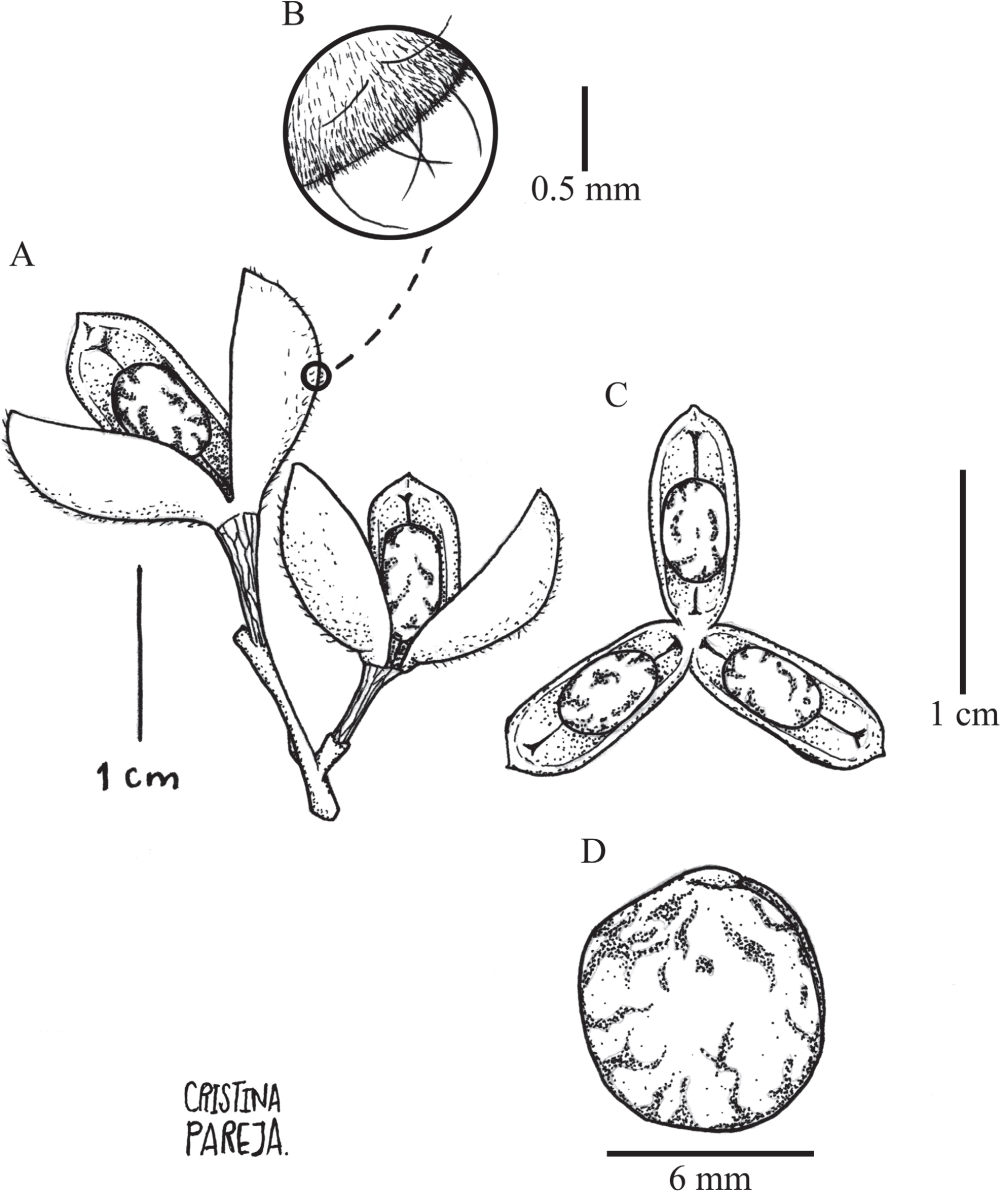
*Rinoreadeflexa* (Benth.) S.F. Blake **A** twig with fruits showing one seed per valve **B** detail of pubescence on fruit surface **C** detail of fruit showing dehiscence with one seed per valve **D** seed, glabrous with maculae (**A–D**: *C. E. Cerón Martínez 18340* [MO]).

Treelet 2–6 m, branchlets sparsely pubescent, glabrescent. Leaves opposite, petiolate; petioles 2–6 cm long, pubescent when young, glabrescent; stipules deciduous, free, linear, 1.9–2.3 × 0.1–0.3 mm, pubescent with trichomes golden, apex mucronulate; lamina elliptic, 4.5–10 × 2.5–5.5 cm, foliaceous, glabrous on both sides, costa pubescent abaxially with golden trichomes, with 8 to 9 major secondary vein pairs, tertiary venation reticulate, base symmetrical, rounded, margin crenate, apex acuminate, acumen 0.1–0.5 cm long, mucronulate, domatia present. Inflorescences axillary and lateral, racemose, solitary, 3–6 × 1–2 cm, flowers dense near the apex, lax near the base, central axis golden brown, pubescent; pedicels 2–2.5 mm long, pubescent with golden trichomes, articulated near the base; bracts, triangular, 1–1.5 × 1–1.5 mm, densely pubescent, trichomes adpressed, 0.2–0.3 mm, golden, ciliolate margin, apex acuminate, mucronulate; bracteoles, triangular, 0.5–1 × 0.5–1 mm, densely pubescent, trichomes adpressed, 0.2–0.3 mm, golden, ciliolate margin, apex acuminate, mucronulate. Flower buds erect to the apex of the inflorescence, becoming strongly deflexed when flowering; sepals subequal, ovate, 1.5–2 × 0.75–1.3 mm, carnose near the base, scarious near the margin, densely pubescent with golden trichomes, mainly along the costa and near the apex, margin ciliate, apex obtuse; petals yellow, narrowly elliptic, tapering to the apex, 3–3.5 × 0.6–0.8 mm, costa pubescent with golden trichomes, margin ciliate, apex obtuse; stamens 2.7–3.5 mm long, filaments free, 0.5–0.8 mm long, dorsal glands 0.5–0.7 mm long, covering the filament, carnose, glabrous, anthers ovoid, 1.2–1.3 × 0.3–0.4 mm, glabrous, connective narrowly deltoid, 0.6–0.9 mm long, glabrous, dorsal anther connective scale lanceolate, 2.2–2.8 × 0.6–0.8 mm, scarious, brownish, margin erose near the apex; ovary globose 0.8–1 cm in diam., pubescent with golden trichomes, style filiform, erect, 2–2.5 mm long, glabrous, stigma apiculate. Fruit a symmetrical subligneous capsule dehiscent along three sutures, born erect at maturity, ellipsoid, 12–16 × 10–14 mm, apex rounded, green at maturity, pubescence dimorphic with golden to ferruginous, small dense trichomes 0.1–0.2 mm long and longer dispersed trichomes 0.3–0.5 mm long, surface veined. Seeds one per valve, globose, 5–6 mm, glabrous, brown, with maculae (Fig. 7).

##### Distribution and ecology.

**Figure 8.**
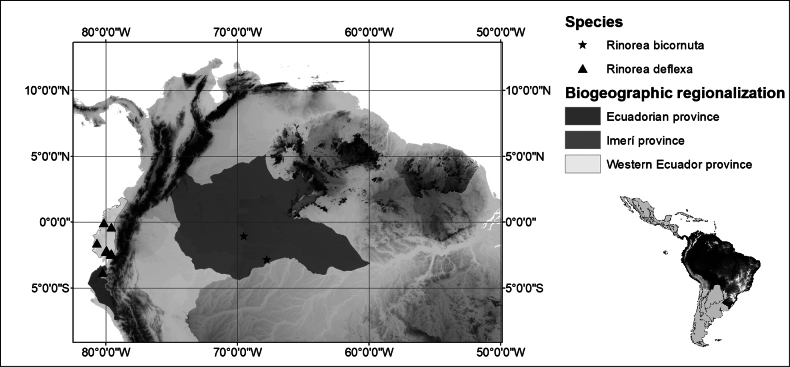
Distribution of *Rinoreabicornuta* (stars) and *R.deflexa* (triangles).

*Rinoreadeflexa* has a restricted distribution in southern Ecuador and northern Peru, in the Western Ecuadorian and Ecuadorian biogeographical provinces (sensu Morrone 2014; Fig. 8). The species grows at elevations of 50–720 m, and it occurs in estuarine forest near riverbanks and in primary and secondary lowland dry forests.

##### Phenology.

The species flowers in January and fruits in January through March.

##### Notes.

*Alsodeiadeflexa* was described by Bentham based on a single flowering specimen. Over the course of revising the genus from Colombia, many additional fruiting herbarium specimens from Ecuador were observed and allowed us to provide an updated description.

##### Additional specimens examined.

**Ecuador. Prov. Guayas**: • Guayas, Naranjal, 02°27'S, 79°38'W, 350 m, 28 Dec 1991 (st), *C. E. Cerón Martínez 17807* (MO); • *ibid*., 250–350 m, 28 Feb 1992 (st), *C. E. Cerón Martínez 18340* (MO); • *ibid*., 02°24'S, 79°36'W, 350–380 m, 11 Aug 1992 (st), *C. E. Cerón Martínez 20069* (MO); • *ibid*., 340–370 m, 17 Aug 1992 (st), *C. E. Cerón Martínez 20331* (MO); • *ibid*., 02°27'S, 79°38'W, 50–70 m, 27 Sep 1992 (st), *C. E. Cerón Martínez 20389* (MO); • *ibid*., 150–200 m, 2 Oct 1992 (st), *C. E. Cerón Martínez 20621* (MO); • Guayas, 02°27'S, 79°36'W, 180 m, 21 Mar 2006 (fr), *T. B. Croat et al. 96202* (MO); • Cerro Azul, 02°15'S, 80°00'W, 50–300 m, 17 Jan 1991 (fl), *A. H. Gentry et al. 72346* (ECUAMZ, MO, JAUM); • Guayas, 12 km from Guayaquil, 18 Feb 1962 (fr), *A. J. Gilmartin 590* (US); • Cerro Blanco, 02°10'S, 79°58'W, 370 m, 27 Feb 1996 (fr), *D. A. Neill et al. 10449* (MO, QNCE); • *ibid*., 02°10'S, 79°57'W, 420 m, 29 Feb 1996 (fr), *D. A. Neill et al. 10495* (MO, QNCE); • Cerro Azul, 02°10'S, 79°58'W, 500 m, 18 Mar 1992 (fr), *W. A. Palacios et al. 9983* (MO); • Guayas, Guayaquil, 02°10'S, 79°58'W, 400 m, 21–25 Jan 1992 (fl), *D. Rubio et al. 2319* (ECUAMZ, MO, QNCE); • *ibid*., (fr), *D. Rubio et al. 2367* (ECUAMZ, MO, QNCE). **Prov. Loja**: • Lajas, 03°53'S, 80°04'W, 350 m, 8 Jun 1995 (fl), *X. Cornejo et al. 4139* (U). **Prov. Manabí**: • estero Perro Muerto, 01°36'S, 80°42'W, 400–420 m, 23 Jan 1991 (st), *A. H. Gentry et al. 72673* (F, MO); • Jama, 0°04'S, 80°09'W, 50 m, 6 Jan 2006 (fl), *D. A. Neill 15107* (MO, QNCE). **Peru**. **Dept. Tumbes**: • Zarumilla, 03°50'S, 80°15'W, 720 m, 25 Jul 1992 (st), *C. Díaz de la Guardia Guerrero 5127A* (MO); • Zarumilla, Campo Verde el Caucho, 03°50'S, 80°15'W, 500 m, 12 Nov 1992 (st), *C. Díaz Santibáñez et al. 6038* (MO); • *ibid*., 12 Nov 1992 (st), *C. Díaz Santibáñez et al. 6087* (MO); • *ibid*., 12 Nov 1992 (st), *C. Díaz Santibáñez et al. 6088* (MO); • *ibid*., 12 Nov 1992 (st), *C. Díaz Santibáñez et al. 6089* (MO); • *ibid*., 10 Feb 1993 (fr), *C. Díaz Santibáñez et al. 6259* (MO); • *ibid*., 15 Feb 1993 (fr), *C. Díaz Santibáñez et al. 6435* (MO); • *ibid*., 18 Feb 1993 (fr), *C. Díaz Santibáñez et al. 6620* (MO); • *ibid*., 18 Feb 1993 (fr), *C. Díaz Santibáñez et al. 6664* (MO); • *ibid*., 20 Jan 1995 (fr), *C. Díaz Santibáñez et al. 7442* (MO).

#### ﻿9. Rinorea bicornuta

Taxon classificationPlantaeMalpighialesViolaceae

Hekking. Phytologia 43: 474. 1979.

FA3F89B3-55CF-50FD-A3B7-14CD504BFB8D

##### Type.

Brazil • Amazonas: Tocantins, Solimões, 1927, *W. A. Ducke 21353* (holotype: RB [barcode RB00560922]!; isotype: RB [barcode RB00544454]!).

##### Description.

**Figure 9.**
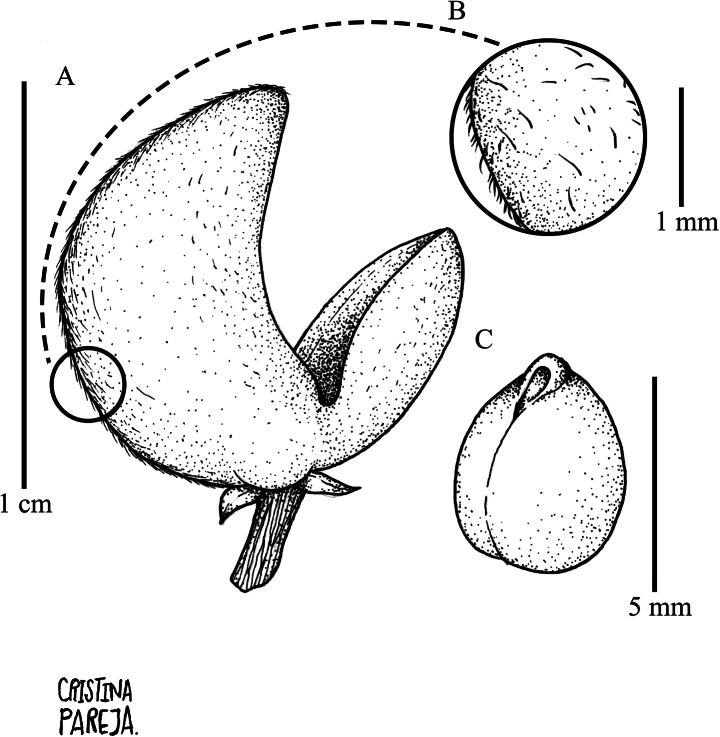
*Rinoreabicornuta* Hekking **A** fruit with asymmetrical valves **B** detail of pubescence on fruit surface **C** seed, glabrous without maculae (**A–C**: *L. Clavijo 404* [HUA]).

Tree 5–12 m tall, branchlets densely strigose with ferruginous erect trichomes 0.2–0.3 mm long, lenticels 0.5–1 mm long. Leaves alternate, petiolate, petioles 6–9 mm long, pilose with golden appressed trichomes 0.3–0.6 mm long, glabrescent; stipules deciduous, free, triangular, 3–4 × 1–1.7 mm, veined, costa pilose, margin ciliate, apex acuminate, mucronulate; lamina elliptic, 10–25 × 5–12 cm, foliaceous, glabrous on both sides, costa sparsely pilose on both sides with golden appressed trichomes 0.1–0.3 mm long, with 8 to 11 pairs of major secondary vein, tertiary venation brochidodromous, base symmetrical, cuneate; margin subentire to subcrenate, apex acuminate, acumen 0.5–1 cm long, mucronulate, domatia absent. Inflorescence axillary and terminal, thyrsoid, solitary or in 2 to 3 fascicles, 7–15 cm long, rachis green when fresh, brown when dry, cymules with 1 to 5 flowers; common peduncle 2.5–5 mm long, pubescent with dispersed pilose golden trichomes 0.1–0.2 mm long; pedicels 2–4 mm long, articulated near the base, pubescent with pilose golden trichomes 0.1–0.2 mm long; bracts deciduous, lanceolate, 1–1.5 × 1–1.5 mm, costa pubescent with golden pilose trichomes 0.1–0.2 mm long, margin ciliate, mucronulate; bracteoles deciduous, ovate, 0.8–1.1 × 0.4–0.6 mm, subopposite, costa pubescent with golden pilose trichomes 0.1–0.2 mm long, margin ciliate, apex acuminate, mucronulate. Flowers subzygomorphic, 3.5–4 × 3.5–4 mm, flower buds conical; sepals ovate, 1.5–2 × 0.9–1.1 mm, pubescent near the base and along the costa with pilose golden trichomes 0.1–0.2 mm long, margin ciliate, apex subobtuse, mucronate, with 1–3 longitudinal veins; petals cream-colored, ovate, 3–4 × 0.8–1.5 mm, pubescent along the costa with dispersed pilose golden trichomes 0.1–0.2 mm long, margin sparsely ciliate, apex obtuse, cream when fresh, brown when dry; stamens 2.5–3 × 1.0–1.2 mm, filaments and dorsal glands adnate to form a staminal tube 0.2 mm tall, carnose, glabrous, anthers 0.8–1 × 0.4–0.5 mm, subsessile, ovoid, with two apical cusps 0.3–0.5 × 0.1–0.2 mm; connective ca. 0.75 × ca. 0.25 mm, pubescent with dispersed pilose trichomes 0.1–0.2 mm long, dorsal anther connective scale lanceolate, 2–2.5 × 0.7–0.9 mm, scarious, margin entire, apex acuminate, brown when dry; ovary 0.8–1 mm, ovate, pubescent apically with pilose golden trichomes 0.2–0.3 mm long; style 2–2.5 mm long, erect, glabrous, stigma apiculate. Fruit an asymmetrical capsule dehiscent along three sutures, ellipsoid, 5–12 mm × 4–8 mm, pubescent with dimorphic golden trichomes, smaller trichomes 0.1 mm long, erect, larger trichomes 0.2–0.4 mm long, appressed; valves unequal, large valve 10–12 × 5–6 mm, smaller valves 6–7 × 4–5 mm, with persistent floral parts at the base. Seeds one per valve, ovoid, 4–5 mm diam., glabrous, shiny brown without maculae, with a conspicuous lateral hilum (Fig. 9).

##### Distribution and habitat.

*Rinoreabicornuta* is an endemic species near the border region of Colombia and Brazil, an area corresponding to the Imerí biogeographical province (sensu Morrone 2014; Fig. 8). It occurs in primary lowland wet forests between 50 and 200 m elevation.

##### Phenology.

The species was recorded in flower in February and November and in fruit in February.

##### Notes.

*Rinoreabicornuta* was described by Hekking (1979) from a single flowering specimen. Over the course of revising the genus from Colombia, a second fruiting herbarium specimen from Vaupés Department was observed and allowed us to provide and updated description here.

##### Additional specimens examined.

Colombia. Vaupés: • Taraira, Estación Biológica Mosiro Itajura (Caparú), 01°04'21.8"S, 69°31'2.9"W, 200 m, 20 Feb 2004 (fl, fr), *L. Clavijo 404* (COAH, COL, HUA, UBDC).

## Supplementary Material

XML Treatment for Rinorea cardenasii

XML Treatment for Rinorea idarragae

XML Treatment for Rinorea pabongonzaleziorum

XML Treatment for Rinorea fernandeziana

XML Treatment for Rinorea laurifolia

XML Treatment for Rinorea ulmifolia

XML Treatment for Rinorea squamata

XML Treatment for Rinorea deflexa

XML Treatment for Rinorea bicornuta

## ﻿Additional information

### Author ORCIDs

Saúl E. Hoyos-Gómez https://orcid.org/0000-0001-7068-6667

Harvey E. Ballard Jr. https://orcid.org/0000-0003-2396-784X

Ricardo Callejas Posada https://orcid.org/0000-0001-6333-5682

Gregory A. Wahlert https://orcid.org/0000-0003-1703-9860

### Data availability

All of the data that support the findings of this study are available in the main text.
